# The use and misuse of procalcitonin in the management of pneumonia: a retrospective analysis at a large tertiary care center

**DOI:** 10.1017/ash.2025.175

**Published:** 2025-05-19

**Authors:** Doris C. Obimba, Aaron Shaykevich, Danielle R. Vitale, Christopher A. Rudmann, Heather Korrie, Joseph Miles, Christopher Noel, Ivayla I. Geneva

**Affiliations:** 1 State University of New York Upstate Medical University, Syracuse, NY, USA; 2 Crouse Hospital, Syracuse, NY, USA

## Abstract

**Objective:**

Antibiotics overuse leads to bacterial resistance. The biomarker procalcitonin rises with bacterial pneumonias and remains normal in viral respiratory tract infections. Its use can distinguish between these etiologies and thus guide antibiotics use. We aimed to quantify the effect of procalcitonin use on clinical decision-making.

**Design:**

A retrospective study, spanning a year at a tertiary care center, where 348 patients hospitalized with aspiration pneumonia and 824 with non-aspiration pneumonia were evaluated with regards to procalcitonin use, the length of stay (LOS) and antibiotics prescribing practices. Descriptive statistics and univariate analyses were applied to the ensemble data. Subsets of cases were manually reviewed and analyzed with descriptive statistics. *P* < 0.05 indicated statistical significance.

**Results:**

21% of both the aspiration and non-aspiration pneumonia cases had procalcitonin checked. In the ensemble analyses, a check of procalcitonin was more likely to happen in prolonged hospitalizations with aspiration pneumonia. The LOS was statistically the same regardless of procalcitonin results (elevated or normal) for both the aspiration and non-aspiration pneumonia cohorts. The overall use of antibiotics was not affected by the procalcitonin results. After excluding two extreme outliers, the per-person antibiotics cost was not affected by the procalcitonin results. Detailed chart reviews of 33 cases revealed that for the vast majority, the procalcitonin results were not used by clinicians to guide the duration of antibiotics use.

**Conclusions:**

Despite its promise as a biomarker for antibiotics stewardship, procalcitonin results appeared to not be utilized by clinicians as a decision-making tool in the management of pneumonia.

## Introduction

The overuse of antibiotics has been associated with the development of antibiotic resistance and is a driver for increase in health care costs.^1,2^ A promising target for antibiotic stewardship is the management of pneumonia. For instance, an aspiration event can result in aspiration pneumonitis without bacterial infection or in bacterial aspiration pneumonia. While both cases can cause fever, dyspnea, and leukocytosis, antibiotics are beneficial only in the latter. Yet, distinguishing between these two cases remains a clinical challenge. Similarly, the differentiation between viral pneumonia and non-aspiration bacterial pneumonia is elusive. The biomarker procalcitonin may provide a solution to both problems.^3–12^ Procalcitonin levels typically rise within 2–4 hours following bacterial infection and peak at 24–48 hours. Subsequently, the procalcitonin levels decrease as the infection is successfully treated. This variation in procalcitonin levels can therefore serve as a tool to help clinicians decide whether the patient has a bacterial infection, whether they are responding to the antibiotics selection, and thus guide antibiotic stewardship.^13,14^ Nevertheless, there is an ongoing controversy regarding the usefulness of procalcitonin in reducing unnecessary antibiotics use, with some US-based studies showing promise^15–17^ while others failed to do so.^18–20^ Additionally, most studies comparing aspiration pneumonia and aspiration pneumonitis have focused on critically ill patients,^5,7,8^ where withholding antibiotics even in the setting of normal procalcitonin levels is unlikely given the illness severity. As such, procalcitonin was found to be mainly useful for determining antibiotics duration among the critically ill. The only non-ICU study of procalcitonin^4^ had predetermined patient groups for aspiration pneumonitis and aspiration pneumonia, demonstrating significantly lower procalcitonin level in the former group—an encouraging result. It should also be noted that procalcitonin testing can result in a significant financial burden for a hospital if it is widely used at the time of antibiotics initiation and during treatment to monitor the response to antibiotics. Our study evaluated the usefulness of procalcitonin in the management of pneumonia at a tertiary medical center with a focus on cost-benefit analysis and healthcare providers’ decision-making. Currently, our hospital does not have a procalcitonin-based antibiotic stewardship policy in place and this study is expected to guide its development.

## Methods

### Ensemble data

**Figure 1.**
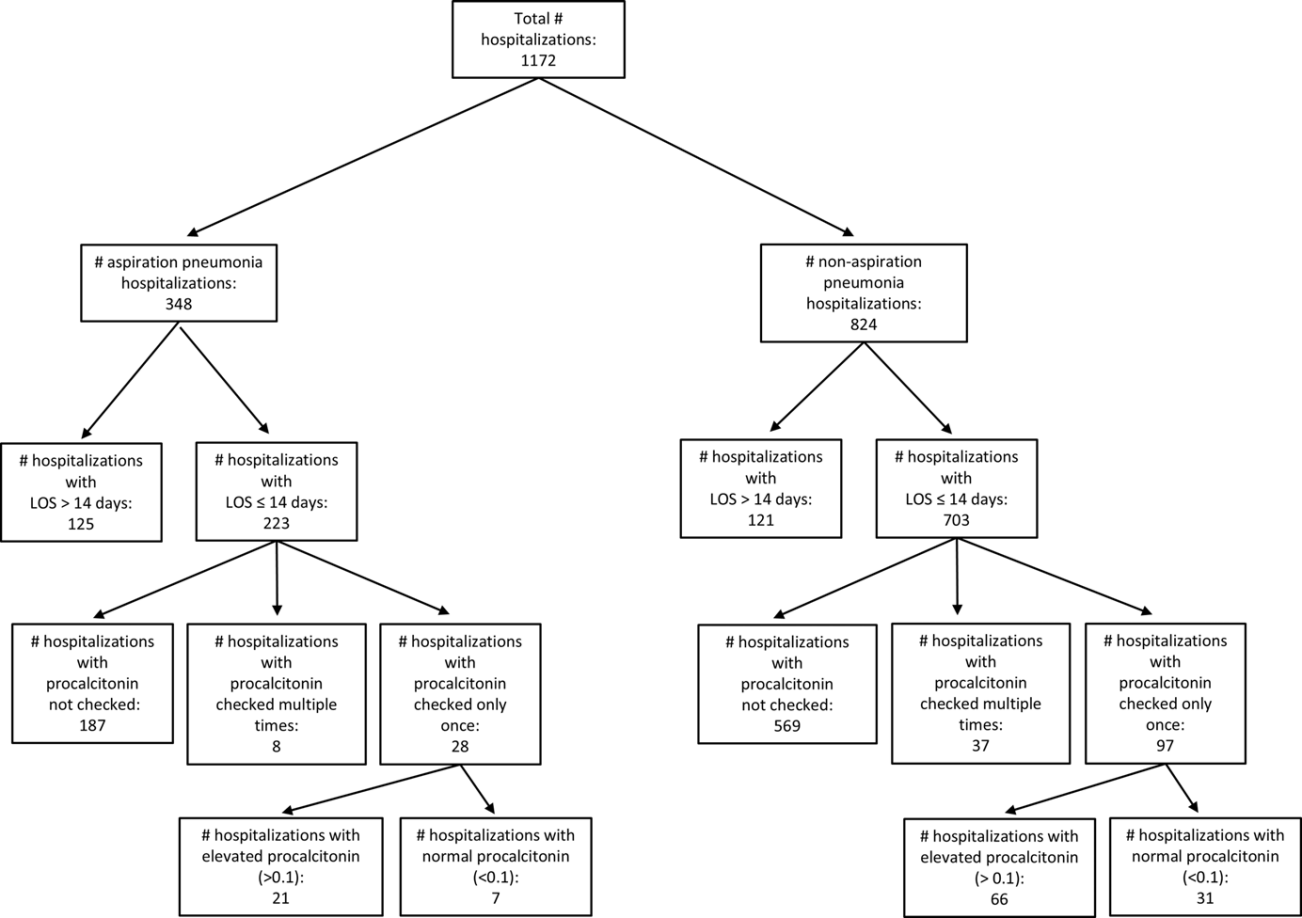
Flowchart for patients included in the ensemble analysis of this study.

This is a retrospective study of patients hospitalized at a tertiary medical center in the USA. Two groups of adults (age ≥18 years) were identified from January 1^st^ 2023 to December 31^st^ 2023 (refer to Figure 1). Group 1 carried the diagnosis of “aspiration pneumonia/pneumonitis” during the hospitalization (ICD10 code J69.0). Group 2 had been treated for non-aspiration pneumonia (ICD10 codes J18, J18.0, J18.1, J18.2, J18.8, J18.9, while excluding J69.0). We automatically extracted the following information from the database: age, length of stay (LOS), procalcitonin testing results (if any), ICD10 diagnoses, number units of each antibiotic used for pneumonia.

For the ensemble analysis, we evaluated the aspiration pneumonia and the non-aspiration pneumonia cohorts separately because they featured significant LOS difference and the bacterial infection etiology was different between the two. Further subgrouping of non-aspiration pneumonia into e.g. community-acquired pneumonia and hospital-associated pneumonia would have required manual chart review of hundreds of hospitalizations, which was deemed unfeasible for our study.

For the univariate analyses, we only included patients with LOS ≤14 days because first, antibiotics courses for the vast majority of pneumonia patients are <14 days; second, from sample manual chart reviews we noticed that patients with longer hospitalization tended to have additional infections requiring longer antibiotics courses, which would have biased our study. For the included patients, we search for additional common infections documented by ICD10 codes and their derivatives—cystitis (N30), cellulitis (L03), meningitis (A39.01, B37.5, G00.9, G02, G03.9), cholecystitis (K81), intestinal infections (A00–A09), and bacteremia (R78.81). The hospitalizations with additional infections were manually reviewed to determine whether these additional infections led to longer antibiotics courses than the pneumonia treatment and whether they triggered the procalcitonin check.

We performed descriptive statistics and univariate analyses. In the latter, regarding procalcitonin level (elevated versus normal), only patients with a single procalcitonin lab per hospitalization were included to avoid the confounding. LOS and age were found to be non-parametric. Mann-Whitney *U* test and χ^2^ Test were used to evaluate the effect of procalcitonin use on the LOS and the use of antibiotics. In the Mann-Whitney *U* test the LOS was treated as a continuous variable, while in the χ^2^ Test, the LOS was treated as a categorical variable with categories: 0–2 days, 3–7 days, >7 days. Spearman’s Correlation and Kruskal-Wallis Test were used to evaluate the effect of patients’ age on their LOS, for continuous LOS and categorical LOS, respectively. Multivariate analysis was initially planned but given the univariate statistics results, it was not beneficial to pursue.

Cost analysis was carried out based on the per-unit cost of all antibiotic types used during the hospitalization and whose indication was “pneumonia.”

### Individual chart reviews

Additional patient selection criteria were imposed for the manual chart reviews. We excluded patients with other (non-pneumonia) bacterial infection requiring antibiotics, severe immunosuppression (i.e., ≥20mg daily prednisone equivalent for ≥2 weeks, immunosuppressive biologics, uncontrolled HIV infection with CD4<200, active malignancy, chemotherapy use), need for ICU (intensive care unit) management, patients with another reason to have baseline elevated procalcitonin (e.g., chronic dialysis, metastatic cancer, surgery in the past 7 days), and those transitioned to comfort care before the planned antibiotics course was completed. We aimed to review 10 cases for each of the four groups: aspiration pneumonia with elevated procalcitonin or normal procalcitonin, and non-aspiration pneumonia with elevated procalcitonin or normal procalcitonin. The data variables are provided in Table 1.

**Table 1.** Detailed clinical information from manually reviewed charts

**ASPIRATION PNEUMONIA**									
***Normal Procalcitonin***									
	procalcitonin level	# days after diagnosis procalcitonin was checked	# days antibiotics after procalcitonin was checked	total # days of antibiotics	white blood cell count	Fever?	Oxygen requirements during hospitalization	Oxygen requirements at baseline	relevant positive microbiology results
Case 1	<0.1	1	9	10	normalized quickly	never	4L, normalized quickly	no oxygen needs	positive MRSA nares PCR
Case 2	<0.1	3	2	5	stable at 12 to 14, no trend	never	4L, normalized quickly	2L	none
Case 3	<0.1	6	2	8	always normal	fever only 5 days BEFORE procalcitonin was checked	2L, normalized quickly	no oxygen needs	none
Case 4	<0.1	2	4	6	always normal	never	no oxygen needs	no oxygen needs	none
Case 5	<0.1	0	0	1	always normal	never	4L, normalized quickly	no oxygen needs	none
Case 6	<0.1	4	1	5	always normal	never	no oxygen needs	no oxygen needs	none
***Elevated Procalcitonin***									
Case 1	9.8	0	6	6	normalized by day 1	resolved by day 1	2L, normalized quickly	no oxygen needs	none
Case 2	4.51	0	9	9	decreased to 13 (from 34) by day 9	resolved by day 1	4L, normalized quickly	no oxygen needs	none
Case 3	3.22	0	7	7	remained elevated	resolved by day 1	10L, decreased over time	no oxygen needs	none
Case 4	13.59	1	10 / 3	11	normalized by day 3	never	15L, decreased over time	no oxygen needs	none
Case 5	13.24 / 0.5	1 / 8	5	7	stable at 12, no trend	resolved by day 1	15L, decreased over time	no oxygen needs	none
Case 6	1.42	2	3	5	stable at 11, no trend	resolved by day 1	4L, normalized quickly	no oxygen needs	none
Case 7	0.41	0	7	7	normalized by day 3	never	15L, decreased quickly	no oxygen needs	none
**NON-ASPIRATION PNEUMONIA**									
***Normal Procalcitonin***									
Case 1	<0.1	-2	6	8	always normal	never	no oxygen needs	no oxygen needs	none
Case 2	<0.1	3	5	8	stable at 12–15, no trend	never	no oxygen needs	no oxygen needs	none
Case 3	<0.1	1	2	3	always normal	never	4L, normalized quickly	no oxygen needs	none
Case 4	<0.1	5	6	11	always normal	never	15L, decreased over time	no oxygen needs	none
Case 5	<0.1	8	0	8	stable at 15, no trend	never	15L, decreased over time	no oxygen needs	none
Case 6	<0.1	2	3	5	always normal	never	2L, normalized quickly	no oxygen needs	none
Case 7	<0.1	0	7	7	normalized by day 2	never	3L, normalized quickly	no oxygen needs	none
Case 8	<0.1	-4	6	10	always normal	never	2L, normalized quickly	no oxygen needs	none
Case 9	<0.1	2	5	7	always normal	never	no oxygen needs	no oxygen needs	none
Case 10	<0.1	0	7	7	normal initially, then increased due to steroids use, then normalized	never	15L, normalized quickly	no oxygen needs	none
***Elevated Procalcitonin***									
Case 1	0.85	2	8	10	at 11 on admission, normalized on day 4	normalized by day 4	2L, normalized quickly	no oxygen needs	none
Case 2	0.67	2	6	8	increased then normalized by day 8	never	4L, normalized quickly	no oxygen needs	none
Case 3	15.18	5	4	9	at 11 on admission, normalized on day 7	never	4L, normalized quickly	no oxygen needs	none
Case 4	1.81	2	9	11	always normal	normalized by day 2	3L, normalized quickly	no oxygen needs	none
Case 5	0.26	1	7	8	at 10.5 on admission, normalized on day 1	never	6L, deceased over time	no oxygen needs	none
Case 6	0.22	0	8	8	always normal	never	60L, decreased over time	4L	none
Case 7	19.78	2	5	7	at 10.9 on admission, normalized on day 1	never	2L, normalized quickly	no oxygen needs	none
Case 8	3.73	2	5	7	at 15.3 on admission, normalized on day 2	never	no oxygen needs	no oxygen needs	none
Case 9	0.38	2	4	6	stable at 11 to 15, no trend	never	no oxygen needs	no oxygen needs	positive COVID-19 PCR
Case 10	0.67	1	10	11	at 14.8 on admission, normalized on day 3	never	no oxygen needs	no oxygen needs	positive Streptococcus pneumoniae urine antigen

## Results

### Descriptive statistics

There were 1,172 hospitalizations with pneumonia and Figure 1 features the patient selection flow. Of those, 348 hospitalizations featured aspiration pneumonia, 44.5% female, average age 72.7 (SD 16.1) (females), 70.9 (SD 14.5) (males). 824 hospitalizations featured non-aspiration pneumonia, (50.4%) female, average age of 70.7 (SD 16.2) (females), 69.5 (SD 16.5) (males). Twenty-one percent of both the aspiration (74 cases) and non-aspiration (175) cohorts had procalcitonin checked.

The LOS for the whole aspiration pneumonia cohort (348 hospitalizations) was on average 20.6 days (SD 45.5) days. When only including hospitalizations of LOS ≤14 days, the average (LOS) was 7.1 (SD 3.8) days. There were 14 cases of additional infections besides pneumonia. The LOS for all non-aspiration pneumonia hospitalizations (824) was 8.7 (SD 9.7) days. For those with LOS ≤14 days, it became 5.7 (SD 3.5) days. There were 29 cases with additional infections. Based on manual chart review of the additional infections, none was the reason for procalcitonin check and only one bacteremia had resulted in longer antibiotics course.

All patients in our study had received antibiotics. When including all 348 hospitalizations, the aspiration pneumonia patients utilized 30 (SD 89) doses, while the 824 non-aspiration pneumonia cases averaged 15 (SD 48). Using the cutoff of LOS ≤14 days, we calculated the following averages: 13.9 (SD 11.7) antibiotics units for aspiration pneumonia cases with normal procalcitonin, 9.2 (SD 10.1) for aspiration pneumonia cases with elevated procalcitonin, 14.1 (SD 23.3) antibiotics units for non-aspiration pneumonia cases with normal procalcitonin, and 10.9 (SD 11.8) for non-aspiration pneumonia cases with elevated procalcitonin.

### Ensemble analysis of procalcitonin’s impact on pneumonia management

Patients with a LOS of ≤14 days were included given that antibiotics courses for pneumonia rarely exceed 14 days and to avoid bias from additional infectious processes besides pneumonia. Aspiration pneumonia and non-aspiration pneumonia hospitalizations were evaluated separately because the LOS was statistically longer for aspiration pneumonia (*P* < 0.05) overall and in all subgroups. We found that patients’ age did not affect the LOS (*P* > 0.05 for all) in neither the aspiration nor the non-aspiration pneumonia cohorts.

χ^2^ correlation showed that for non-aspiration pneumonia cohort, the LOS was associated with the use of procalcitonin (*P* = 0.001), with longer LOS having higher percentage of procalcitonin checked as follows: 13.2% had procalcitonin checked among those staying 0–2 days, 17.2% for the 3–7 days cohort, and 29.2% for the >7 days group. Similar testing did not yield significant results for the aspiration pneumonia cohort (*P* = 0.105). Yet, there seemed to be a trend towards more procalcitonin testing as the LOS increased 11.1% had it checked from the cases with an LOS of 0–2 days, (9.3%) from the 3–7 days LOS cases, and 20.2% from the >8 days LOS cases. Interestingly, for both aspiration (Mann-Whitney U Test *P* = 0.113; χ^2^ Test *P* = 0.083) and non-aspiration pneumonia cases (Mann-Whitney U Test *P* = 0.988; χ^2^ Test *P* = 0.644), the LOS of patients with elevated procalcitonin was not different from the LOS of patents with normal procalcitonin.

Further, Mann–Whitney *U* testing revealed that for both aspiration (*P* = 0.303) and non-aspiration (*P* = 0.168) pneumonia cases, the amount of antibiotics units used during the hospitalization was not different when comparing those who had procalcitonin checked and those who did not have it checked. Regarding the value of the measured procalcitonin (normal versus elevated) on antibiotics use, we found no difference for neither aspiration pneumonia (*P* = 0.395) nor for non-aspiration pneumonia (*P* = 0.765).

Although initially planned, multivariate analysis was not advisable given that the univariate statistics did not yield useful variables.

### Evaluation of healthcare providers’ use of procalcitonin via individual case reviews

Table 1 contains the manually extracted clinical information for 33 pneumonia cases. For the non-aspiration pneumonia cohort, we were able to identify 10 with normal and 10 with elevated procalcitonin that met the inclusion and exclusion criteria; for the aspiration pneumonia group only 6 patients with normal procalcitonin and 7 with elevated procalcitonin met the criteria.

All of the aspiration pneumonia cases were started on antibiotics on the day of aspiration. But from among the non-aspiration pneumonia cases, in one case antibiotics were delayed by one day in the setting of normal procalcitonin and in another case antibiotics were delayed by 3 days in the setting of an elevated procalcitonin.

Among the 13 aspiration pneumonia cases, procalcitonin was checked within the first 24 hours of the pneumonia diagnosis in 8 cases, at 48 hours in 2, and after 48 hours in 3. Regarding the 20 non-aspiration pneumonia hospitalizations, 6 had procalcitonin checked within the first 24 hours after the diagnosis of pneumonia was made, 8 had it checked at 48 hours, and 4 after more than 48 hours; there were also two unusual cases: there procalcitonin was checked two days *before* and four days *before* the pneumonia diagnosis was documented, both were <0.1.

Regarding the use of antibiotics, for aspiration pneumonia, the total antibiotics courses ranged from 1 to 10 days (mean of 5.8) for normal procalcitonin, from 5 to 11 days (mean of 7.4) for elevated procalcitonin. For non-aspiration pneumonia, the total antibiotics course ranged from 3 to 11 days (mean of 7.4) for normal procalcitonin and from 6 to 11 days (mean of 8.5) for elevated procalcitonin.

Only a minority of the cases seemed to follow the expected logical sequence. Namely, among the aspiration pneumonia cases featuring normal procalcitonin, in only one case the antibiotics were stopped when the procalcitonin result became available. Another case had the antibiotics stopped after 24 hours. The remaining 4 cases had antibiotics continued for 2 to 9 days (average of 4.5), despite these patients having normal WBCs, no fever, no increased oxygen requirements from baseline or the oxygen requirements had been diminishing.

The evaluated non-aspiration pneumonia cases fared even worse regarding antibiotic stewardship. In only one case the antibiotics were stopped upon receiving the normal procalcitonin result. The rest of the cases had additional antibiotics prescribed for 2 to 7 days (average of 4.7), again despite of evidence for rapid clinical improvement. Regarding the cases with elevated procalcitonin, all patients were given additional antibiotics for 4 to 10 days (average of 6.6).

Regarding microbiological data in the detailed chart reviews, there were very few positive results, although microbiological testing was performed in all patients. Namely, there was one positive MRSA nares PCR screen for a patient with aspiration pneumonia and normal procalcitonin, one case of COVID-19 positive PCR test and one case of positive Streptococcal urine antigen test among the non-aspiration pneumonia cases with elevated procalcitonin.

### Cost – benefits analysis

The cost associated with procalcitonin serum testing was $16.21 USD (US dollars) per procalcitonin lab, which did not include the cost associated with the blood drawing as this is not separately reported at our hospital. 341 procalcitonin labs were used in our 1,172 pneumonia patients for a total of $5,529 USD.

**Figure 2.**
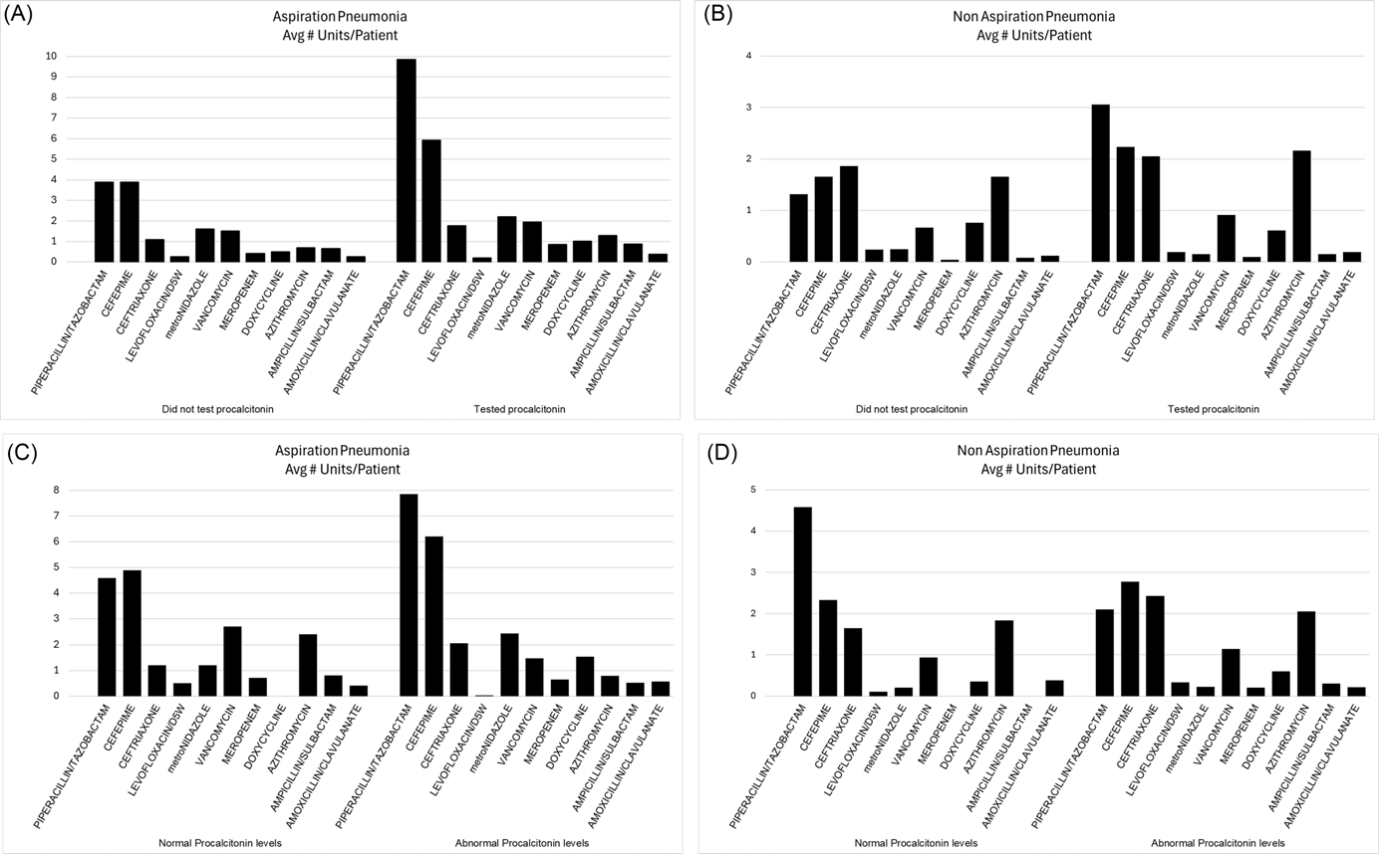
Top 10 antibiotics used for treating pneumonia and the associated per-person average antibiotic costs. (A) Comparing aspiration pneumonia cases without procalcitonin checked versus the cases with procalcitonin results; (B) Comparing aspiration pneumonia cases with normal procalcitonin versus the cases with elevated procalcitonin; (C) Comparing non-aspiration pneumonia cases without procalcitonin checked versus the cases with procalcitonin results; (D) Comparing non-aspiration pneumonia cases with normal procalcitonin versus the cases with elevated procalcitonin.

Regarding antibiotics expenses, Supplemental Table 1 features all antibiotics used for our patients in the form of average number of antibiotic units used per person, the unit cost of the various antibiotics as well as the average per-person total antibiotic costs. Figure 2 illustrates the per-person antibiotics units used during hospitalization for the top-10 antibiotics used in each category (aspiration pneumonias with or without procalcitonin checked, elevated or normal procalcitonin, and similar for the non-aspiration pneumonia patients). Figure 2 also shows the average per-person total antibiotics expenses for each category. In the aspiration pneumonia cohort, the antibiotics cost was statistically higher for those who had procalcitonin checked as compared with those who did not; also, the cost was higher for those with elevated compared to those with normal procalcitonin. However, this difference was caused by two extreme outliers, who used very expensive broad-spectrum antibiotics – ceftazidime-avibactam and cefiderocol, respectively.

Finally, no LOS benefits were noted—the LOS was affected by neither the use of procalcitonin nor the procalcitonin results. This lack of benefits affected both the aspiration and non-aspiration pneumonia cohorts.

## Discussion

Procalcitonin’s applications in antimicrobial stewardship remain controversial, with conflicting studies from both US^15–19^ and European hospitals.^14,21–23^ Our retrospective study of aspiration and non-aspiration pneumonia patients shed light on physician practices at our institutions regarding the use of procalcitonin.

We found that clinicians used procalcitonin more often if the patient stays longer in the hospital, which could help decide whether the antibiotics are working or whether they can be discontinued. However, clinicians did not act appropriately based on the procalcitonin results, i.e. a normal procalcitonin value did not correlate with less antibiotics used, no lower costs (when excluding 2 major outliers), nor shorter LOS.

Further, in the majority (9 out of 13) of the manually reviewed cases of aspiration pneumonia, procalcitonin was checked too early to be of value because it is well-established that it takes 48 hours for a frank bacterial pneumonia to develop following an aspiration event.^13,14^ Therefore, the usefulness of procalcitonin elevation to distinguish aspiration pneumonitis from aspiration bacterial pneumonia is expected to become pronounced starting at 48 hours.

The detailed analysis of individual pneumonia cases mirrored the ensemble data results, where medical providers only rarely stopped the antibiotics in the setting of normal procalcitonin (2 out of 6 cases) in the aspiration pneumonia group and 1 out of 10 cases in the non-aspiration pneumonia group. Certainly, there appeared to be a trend towards longer antibiotics courses when procalcitonin was elevated – 5.8 versus 6.8 days for aspiration pneumonia and 7.4 versus 8.5 days for non-aspiration pneumonia in this small number of cases. However, the ensemble data demonstrated no statistically significant correlation.

Additionally, the detailed analysis of individual cases revealed that the total days of antibiotics use were higher than the antibiotics course recommended by the American Thoracic Society (ATS) and the Infectious Diseases Society of America guidelines (IDSA),^24,25^ which cover both aspiration and non-aspiration pneumonias. According to the guidelines, for patients with community-acquired pneumonias and vital signs stability and clinical improvement, the recommended antibiotics duration is 5 days, and up to 7 days for suspected or proven MRSA or Pseudomonas pneumonia.^24^ For hospital-acquired and ventilator-associated pneumonias the recommendation is 7 days.^25^ Also, in regards to aspiration pneumonia, we noted antibiotics initiation on the same day when aspiration was documented. However, previously published research had demonstrated a lack of clinical benefit of prophylactic antimicrobial treatment in the setting of an acute aspiration event, which may even generate antibiotic selective pressures.^26^

A goal of our retrospective study was to provide a cost-benefit analysis for the use of procalcitonin in the management of pneumonia at our institution. Given the ensemble analyses and the data from the 33 individually reviewed cases, it is quite clear that there is no clinical benefit to be gained through procalcitonin use. While the overall cost associated with running the procalcitonin labs was not large ($5.528 USD over one year), it constituted an unnecessary expense for the hospital.

The limitations of our study include the fact that in the ensemble analysis, we did not have information regarding all possible additional infections, yet the most common ones were identified and accounted for. Further, in the antibiotics analysis, we used antibiotics units (doses) rather than antibiotics days due to lack of the latter data. Our approach was useful for the cost-benefit analysis but may have added undue bias to the antibiotics use in the ensemble analysis. Another potential limitation is the fact that we were not able to subdivide the patients into community-acquired versus hospital-acquired or ventilator-associated pneumonias, which would have been useful as the guidelines for antibiotics duration are different for the two groups, i.e., 5 versus 7 days of antibiotics.

## Conclusion

Our study demonstrated that the use of procalcitonin for the management of pneumonia at our tertiary medical center did not lead to the desirable outcomes of length of stay shortening, decrease in the antibiotics use and antibiotics-related costs. Further, we noted an overuse of antibiotics for bacterial pneumonia based on the recommendations in the ATS-IDSA guidelines. While the cost associated with procalcitonin testing was not overwhelmingly high, it constituted an unnecessary expense that needs to be addressed via educational initiatives targeting medical providers as well as the development of internal policies regarding the use of procalcitonin.
